# Promotility Action of the Probiotic *Bifidobacterium lactis* HN019 Extract Compared with Prucalopride in Isolated Rat Large Intestine

**DOI:** 10.3389/fnins.2017.00020

**Published:** 2017-01-26

**Authors:** Julie E. Dalziel, Rachel C. Anderson, Jason S. Peters, Amy T. Lynch, Nick J. Spencer, James Dekker, Nicole C. Roy

**Affiliations:** ^1^Food Nutrition and Health Team, Food and Bio-based Products Group, AgResearchPalmerston North, New Zealand; ^2^Riddet Institute, Massey UniversityPalmerston North, New Zealand; ^3^Discipline of Human Physiology, Flinders University, School of MedicineAdelaide, SA, Australia; ^4^Fonterra Research and Development CentrePalmerston North, New Zealand

**Keywords:** serotonin agonist, constipation, colon, motility, contraction, enteric nervous system

## Abstract

Attention is increasingly being focussed on probiotics as potential agents to restore or improve gastrointestinal (GI) transit. Determining mechanism of action would support robust health claims. The probiotic bacterium *Bifidobacterium lactis* HN019 reduces transit time, but its mechanisms of action and effects on motility patterns are poorly understood. The aim of this study was to investigate changes in GI motility induced by an extract of HN019 on distinct patterns of colonic motility in isolated rat large intestine, compared with a known promotility modulator, prucalopride. The large intestines from male Sprague Dawley rats (3–6 months) were perfused with Kreb's buffer at 37°C in an oxygenated tissue bath. Isometric force transducers recorded changes in circular muscle activity at four independent locations assessing contractile propagation between the proximal colon and the rectum. HN019 extract was perfused through the tissue bath and differences in tension and frequency quantified relative to pre-treatment controls. Prucalopride (1 μM) increased the frequency of propagating contractions (by 75 ± 26%) in the majority of preparations studied (10/12), concurrently decreasing the frequency of non-propagating contractions (by 50 ± 11%). HN019 extract had no effect on contractile activity during exposure (*n* = 8). However, following wash out, contraction amplitude of propagating contractions increased (by 55 ± 18%) in the distal colon, while the frequency of non-propagating proximal contractions decreased by 57 ± 7%. The prokinetic action of prucalopride increased the frequency of synchronous contractions along the length of colon, likely explaining increased colonic rate of transit *in vivo*. HN019 extract modified motility patterns in a different manner by promoting propagating contractile amplitude and inhibiting non-propagations, also demonstrating prokinetic activity consistent with the reduction of constipation by *B. lactis* HN019 in humans.

## Introduction

Constipation is a common functional gastrointestinal (GI) disorder affecting 20% of the general population worldwide (Vazquez Roque and Bouras, 2015). As a frequently subclinical undiagnosed condition, preventative therapeutic strategies and treatments using natural products are often sought in preference to pharmaceuticals. Probiotic bacteria are attributed with health promoting properties for improving GI discomfort and there are a growing number of studies supporting alteration of GI motility patterns (Ohashi et al., 2001; Lesniewska et al., 2006; Massi et al., 2006; Wang et al., 2010a,b; Wu et al., 2013; Dalziel et al., 2015).

Beneficial probiotic bacteria have been shown to improve symptoms of GI discomfort through relief of constipation and/or diarrhea in clinical studies (Ringel et al., 2012; Sanders et al., 2013). A meta-analysis of randomized controlled trials found that short-term probiotic supplementation decreases GI transit time in constipated or older adults (Miller and Ouwehand, 2013). For example, *Lactobacillus casei* Shirota has been shown to reduce colonic transit time in female adults, relieving chronic constipation (Krammer et al., 2011), and to reduce antibiotic associated diarrhea (Wong et al., 2014). The probiotic *Bifidobacterium lactis* HN019 reduces total transit time in adults with functional GI constipation when used alone (Waller et al., 2011) or in combination with other probiotic strains (Magro et al., 2014), and decreases the severity of diarrhea in piglets (Shu et al., 2001). Some of these effects may occur indirectly, for example *B. lactis* HN019 supplementation increases the resident bifidobacteria population in feces (Ahmed et al., 2007) and these species are reduced in functional constipation in the elderly (Kim et al., 2015). However, it is unknown whether *B. lactis* HN019 might also act directly on enteric neurons in the large intestine to alter motility patterns and thus influence GI transit of solid contents.

The propulsion of luminal contents is coordinated by synchronized contraction and relaxation of GI smooth muscles that are largely controlled by the enteric nervous system (Spencer et al., 2016). Although serotonin is present at high levels in GI tissue, serotonin neurotransmission is not required for the major colonic motor patterns associated with orderly GI transit (Spencer, 2015). Rather, serotonin receptors located on enteric neurons (intrinsic primary afferent neurons in the submucosal plexus) have a modulatory role in coordinating contractile function and are therefore the target of therapeutic treatments for colonic dysmotility, particularly constipation. The serotonin agonist prucalopride is highly selective for the 5-HT4 receptor subtype and is used to treat severe constipation due to decreased GI motility, by stimulating colonic mass movements which provide the main propulsive force for defecation (Bouras et al., 1999). Prucalopride is known to increase the frequency of colonic contractions in the isolated rat large intestine (Yu et al., 2015) and we have recently shown that prucalopride increases colonic transit of solids *in vivo* (Dalziel et al., 2016). As a promotility modulator with a specific mode of action, prucalopride is considered a benchmark compound to compare against substances with an unknown mechanism of action.

The aim of this research was to characterize and quantify changes in GI motility patterns underlying propulsion of luminal contents due to the probiotic bacterium *B. lactis* HN019, which is known to reduce constipation in humans (Waller et al., 2011). We hypothesized that the reduced constipation effect of HN019 is attributed to enhanced synchronous contractions in the colon. We used an *ex vivo* rat model of colonic motility because this provides a well-accepted model for human GI motility studies (Dalziel et al., 2014, 2015) and motility patterns that propel contents are well described for this species (Chen et al., 2013; Costa et al., 2013). We compared the effect of HN019 on motility patterns with that of prucalopride.

We have found bacterial extracts (Dalziel et al., 2015) to be effective at altering motility in this model, as have other *in vitro* studies using bacterial extract from different sources (Massi et al., 2006; Bar et al., 2009). We examined the effect of prucalopride and *B. lactis* HN019 on contractile amplitude and frequency of spontaneous muscle contractions in the isolated rat colon. The pattern of contractions was studied by comparing the probability that a contraction occurring in the proximal colon would fully propagate through to the mid-colon, distal colon and rectum and therefore be synchronized in time.

## Methods

### Bacterial extract

A bacterial extract was used in this study as opposed to live bacteria because *B. lactis* HN019 is a facultative anaerobe so would not be compatible with the oxygenated tissue bath used here. While HN109 may survive in the aerobic assay conditions, its metabolism would be greatly altered compared to what would occur in the colon. Facultative anaerobes switch between fermentation and anaerobic respiration depending on whether oxygen is present or not, which results in the production of different sets of metabolites. In contrast to the assay conditions, the human colonic lumen is almost oxygen-free (O_2_ partial pressure < 1 mM Hg) (Espey, 2013). Therefore in this study we chose to grow HN019 in anaerobic conditions so that it would produce the metabolite profile expected in the colon, and then add this anaerobic produced extract to the assay. *B. lactis* HN019 stock cultures were supplied by Fonterra Research & Development Centre. HN019 primary culture was inoculated from a secondary plate into 10 mL of MRS broth (Merck) and incubated at 37°C in an anaerobic workstation (Concept Plus, Ruskin Technology Ltd, UK) containing 10% CO_2_, 10% H_2_ and 80% N_2_ for 48 h. A 50 mL secondary culture was inoculated with 0.5 mL of primary broth (adjusted to an OD_600_ of 1.5) and incubated anaerobically for 16 h. Two 150 mL secondary cultures were inoculated with 1.5 mL of secondary broth and incubated anaerobically for 16 h to the stationary phase. The bacterial cell culture was harvested and processed into extract under anaerobic conditions, then used in the motility assays freshly each day. Bacterial cells were collected by centrifugation (10,845 g for 20 min at 4°C) and resuspended in 5 mL of anaerobic Krebs solution (118 mM NaCl, 4.7 mM KCl, 1.2 mM KH_2_PO_4_, 1.2 mM MgSO_4_, 2.6 mM CaCl_2_, 25 mM NaHCO_3_, 11 mM glucose, pH 7.4). The bacterial cell pellet was washed twice in Krebs solution, resuspended in 5 mL and incubated on ice for 10 min. The mixture was then sonicated on ice using 20 s pulses with 30 s intervals, power level 2 at 40% duty for 10 min (Vibra-Cell, Sonics and Materials, Newtown, USA). The sonicated mixture was then centrifuged (10,845 g for 30 min) to remove the cell debris and the resulting supernatant was ultra-centrifuged to remove any remaining cell membranes (300,000 g for 2 h) and to prevent excessive frothing of the solution in the motility experiment phase. The final supernatant was a cell-free extract (HN019 extract) that was adjusted to pH 7.4 (with 5 M sodium hydroxide) and diluted 1/10 dilution in Kreb's solution for use in the motility assay.

### Animals

This study was carried out in strict accordance with the recommendations of the New Zealand Animal Welfare Act 1999. The protocol was approved by the AgResearch Limited (Grasslands) Animal Ethics Committee (Ethics Approval No.: AE13449). Male adult Sprague Dawley rats, 3–6 months of age, weighing 250–400 g were obtained from AgResearch Ruakura (Hamilton, NZ). The rats were housed under a 12 h light/dark cycle, and fed Sharpes Diet 86 (Sharpes Stockfeeds Ltd., Carterton, New Zealand). Food and water were available *ad libitum*.

### Whole large intestine

The protocol for recording motility in isolated intact whole large intestine has recently been described (Dalziel et al., 2014, 2015). Briefly, following initial isoflurane anesthesia using a drop-box, the animal was maintained on 5% isoflurane via nose cone and placed in dorsal recumbency. A midline laparotomy was performed and the entire large intestine removed and placed immediately in a beaker containing oxygenated Krebs solution to preserve enteric neuron function. The animal then received an intra-cardiac injection of a lethal dose of sodium pentobarbital. The beaker containing the tissue received further carbogen gas (95% O_2_, 5% CO_2_) whilst the colon was gently flushed with Krebs solution to expel fecal pellets and the entire tissue was then mounted in an organ bath (approximately 350 mL capacity), a stainless steel rod (35 cm in length and 2 mm in diameter) was inserted through the lumen of the colon, which was then perfused at 20 mL/min with Krebs buffer at 35 ± 1°C. The lumen was also perfused with Krebs buffer at 1.5 mL/min which was pumped aborally using a constant flow pump because this provided the pressure required to record consistent propagating contractions. Changes in circular muscle tension were recorded from four sites simultaneously along the length of large intestine, using four custom-made metal hooks anchored 3 cm from both oral and anal ends of the preparation and evenly spaced apart at approximately 4 cm intervals. These hooks were connected via silk thread to force transducers and contractions measured after applying 2 g of tension. Muscle contraction data were recorded using isometric force transducers (MLT0201) connected to an eight-channel bridge amplifier, integrated using PowerLab 8/35 hardware and acquired and analyzed using LabChart 8 software. All recording equipment hardware and software were from ADInstruments Pty Ltd., Bella Vista, NSW, Australia.

### Definition criteria for motility patterns—synchronous vs. non-synchronous

“Synchronous contractions” were defined as contractions that were temporally coordinated and occurring at four independent isometric recording sites. It is recognized that their precise direction of propagation cannot be quantified based on the resolution of four recording sites alone (Spencer et al., 2016). This definition would therefore, in theory, include contractions that propagated in either an anterograde (anally migrating), retrograde (orally migrating), or bi-directionally propagating (i.e., starting in the mid colon and propagating both in an anterograde and retrograde direction). Non-synchronous contractions were those occurring in the proximal colon that were not temporally coordinated with more distal recording sites.

Contraction frequency and amplitude were measured during a 30 min control recording and compared with that over 0–30 and 30–60 min of exposure to treatments, and following 60 min of washout. Because these synchronous and non-synchronous contractions are likely play a major role for coordinated propulsion of luminal contents, they were statistically compared between pre-treatment control and treatment groups. All pharmacological agents and HN019 extract were applied to the serosal side of each preparation, via the perfusion tube supplying the bath. Thus, these substances would need to be absorbed and reach the peripheral circulation to be capable of modulating colonic motility.

### Statistical analysis

Results are expressed as the mean ± SEM from 8 to 10 animals. Repeated measures analysis of variance (ANOVA) with one experimental factor (the three treatments) and one repeated factor (four time points) was used to analyze differences in the frequency and amplitude of synchronous contractions within experiments, and also compared with the Krebs treatment control. All analyses were carried out using the R software version 3.2.3. ANOVA assumptions were met through log or square root transformation where necessary. Linear mixed effects models were used with appropriate variance function for modeling heterodasticity.

Data were excluded from statistical analysis where they were contrary to the main effects observed: 2/12 preparations for prucalopride and 2/10 preparations for HN019, and are described in the results section.

## Results

**Figure 1:**
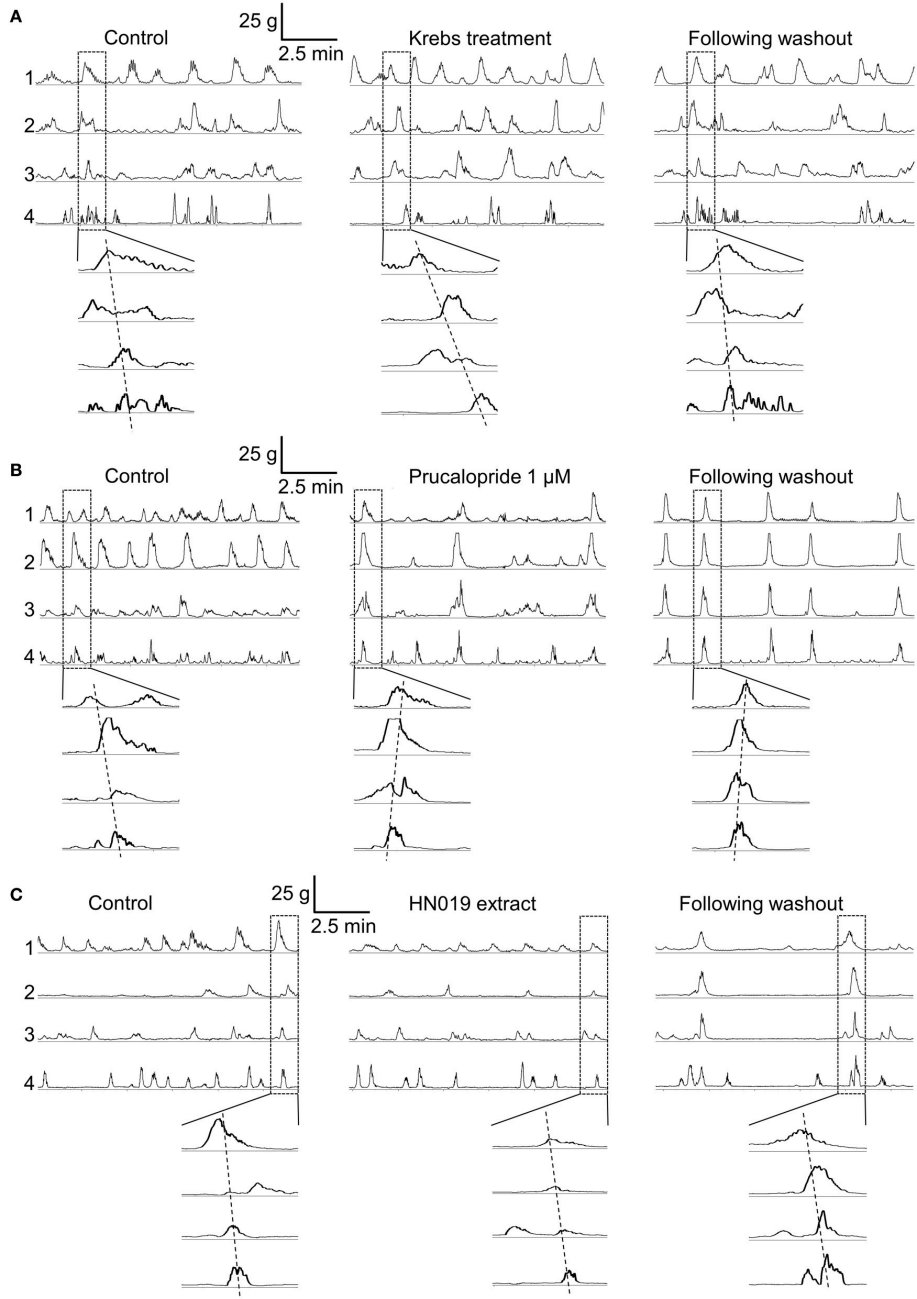
**Contractile motility patterns in the isolated large intestine**. Representative examples show a 10 min recording of muscle contraction from each of the four recording locations for the pre-treatment control (after 1–2 h of equilibration), over 30–60 min of treatment with: **(A)** Krebs buffer treatment control, **(B)** 1 μM prucalopride, and **(C)** HN019 extract (10%), and following 60 min of washout with Krebs buffer. The enlarged windows show synchronous contractions that overlap in time.

Contractile patterns were recorded from the circular muscle layer at four locations simultaneously along the length of the large intestine (proximal colon, mid-colon, distal colon, and rectum), before and after addition of treatment conditions. Motility patterns were quantified with respect to changes in frequency and amplitude of synchronous and non-synchronous phasic contractions, and contractions that occurred only in the proximal colon (Figure 1).

### Krebs treatment control did not alter synchronous contraction patterns

**Figure 2:**
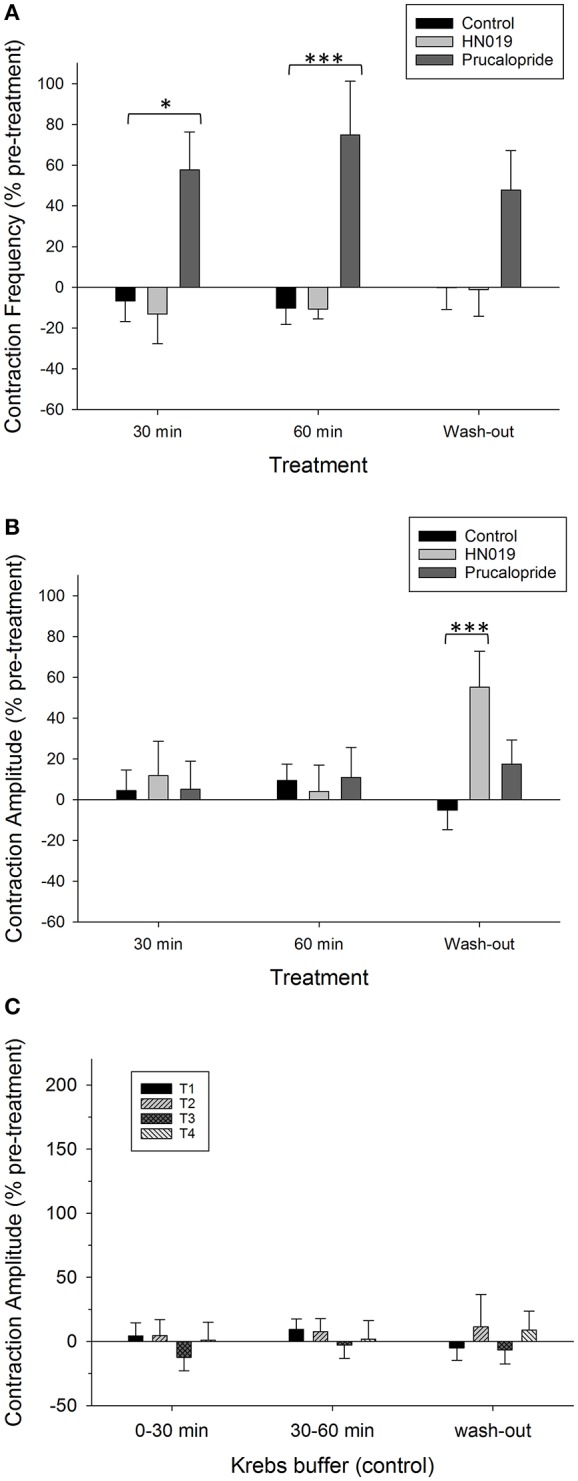
**Summary graphs of treatment effects on synchronous motility parameters in the isolated large intestine**. Data are shown as percent change from the pre-treatment control for: Krebs buffer treatment control (black) (*n* = 8), prucalopride (dark gray) (*n* = 10), and HN019 extract (10%) (light gray) (*n* = 8). Synchronous contractions between the proximal colon and the rectum that were temporally coordinated in anterograde, mid, or retrograde direction were measured during a 30 min control recording and compared with that over 0–30 and 30–60 min of exposure to treatments, and following 60 min of washout. Contraction **(A)** frequency and **(B)** amplitude are shown for the proximal colon. **(C)** The amplitude of synchronous contractions are shown as percent change in contractile amplitude from the pre-treatment control at all four locations (T1, proximal; T2, mid; T3, distal; T4, rectal) for Krebs buffer control treatment condition (*n* = 8), and following 60 min of washout with Krebs buffer. Statistical significance was determined using repeated measures ANOVA and treatment compared with either Krebs buffer treatment control or the pre-treatment control. Asterisks indicate statistical significance (^*^p < 0.05; ^***^p < 0.001). Data show mean ± SEM.

**Figure 3:**
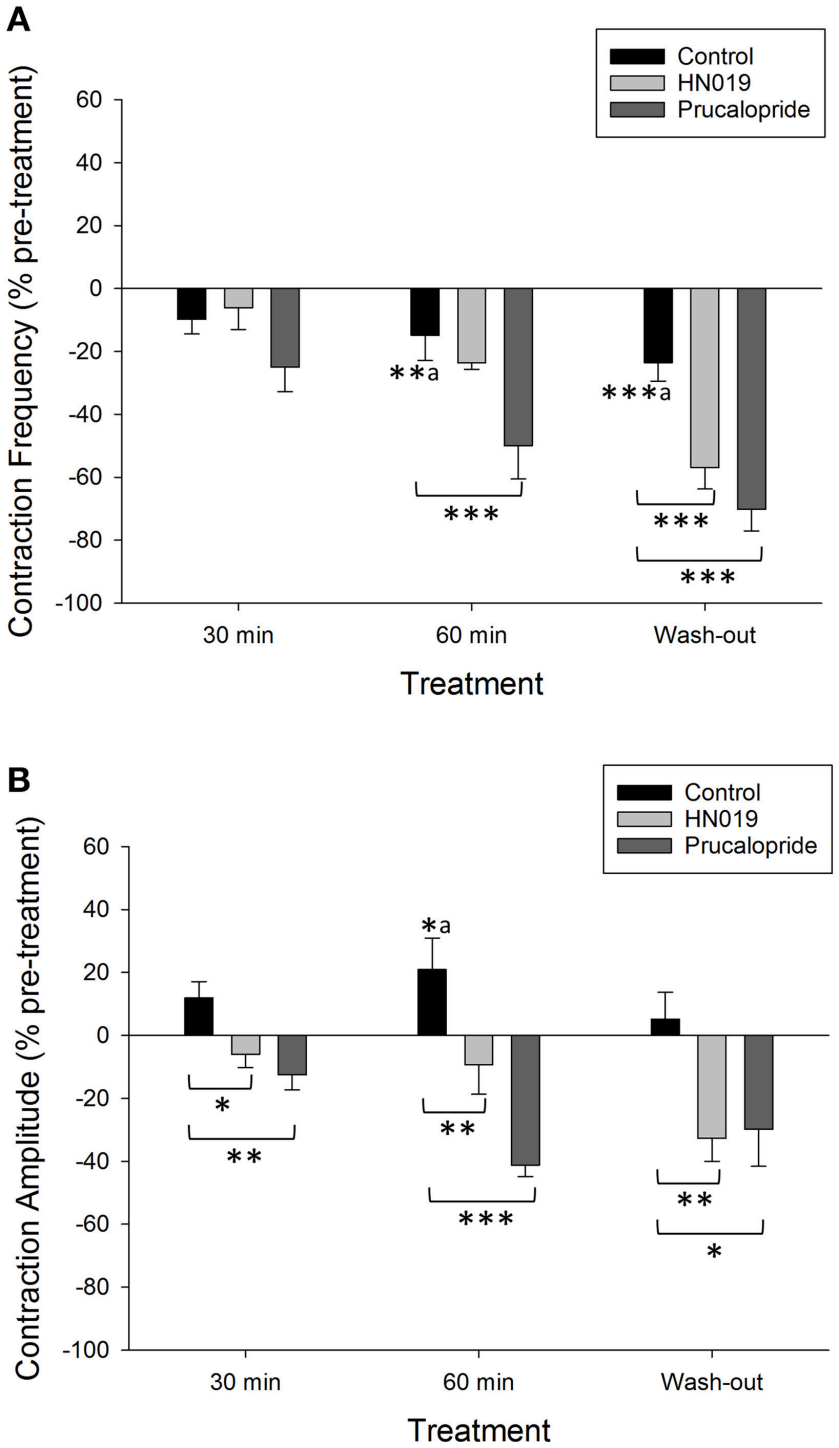
**Summary graphs of treatment effects on non-synchronous motility parameters in the isolated large intestine**. Data are shown as percent change from the pre-treatment control for: Krebs buffer treatment control (black) (*n* = 8), prucalopride (dark gray) (*n* = 10), and HN019 extract (10%) (light gray) (*n* = 8). Non-synchronous contraction **(A)** frequency and **(B)** amplitude, were measured during a 30 min control recording and compared with that over 0–30 and 30–60 min of exposure to treatments, and following 60 min of washout. Amplitude was measured in the proximal colon. Statistical significance was determined using repeated measures ANOVA and treatment compared with either Krebs buffer treatment control or the pre-treatment control (^*a*^ shown only for Krebs). Asterisks indicate statistical significance (^*^p < 0.05; ^**^p < 0.01; ^***^p < 0.001). Data show mean ± SEM.

An initial control experiment was carried out using Krebs buffer to provide a treatment condition, with the aim to ascertain whether any time-dependent changes in contractility occurred and for statistical comparison among treatment groups (Figure 1A). During the 30 min pre-treatment recording, following exposure to Krebs buffer for 1–2 h, 4.4 ± 0.4 (mean ± SEM; *n* = 8) synchronous contractions occurred between proximal colon and rectum. Although no changes in synchronous contractions were detected over the course of the experiment (Figure 2), the frequency of non-synchronous contractions decreased by 15 ± 8% and the peak force generated during non-synchronous contractions increased by 21 ± 10%, over 30–60 min (Figure 3).

### Prucalopride increased the frequency of propagating synchronous contractions

**Figure 4:**
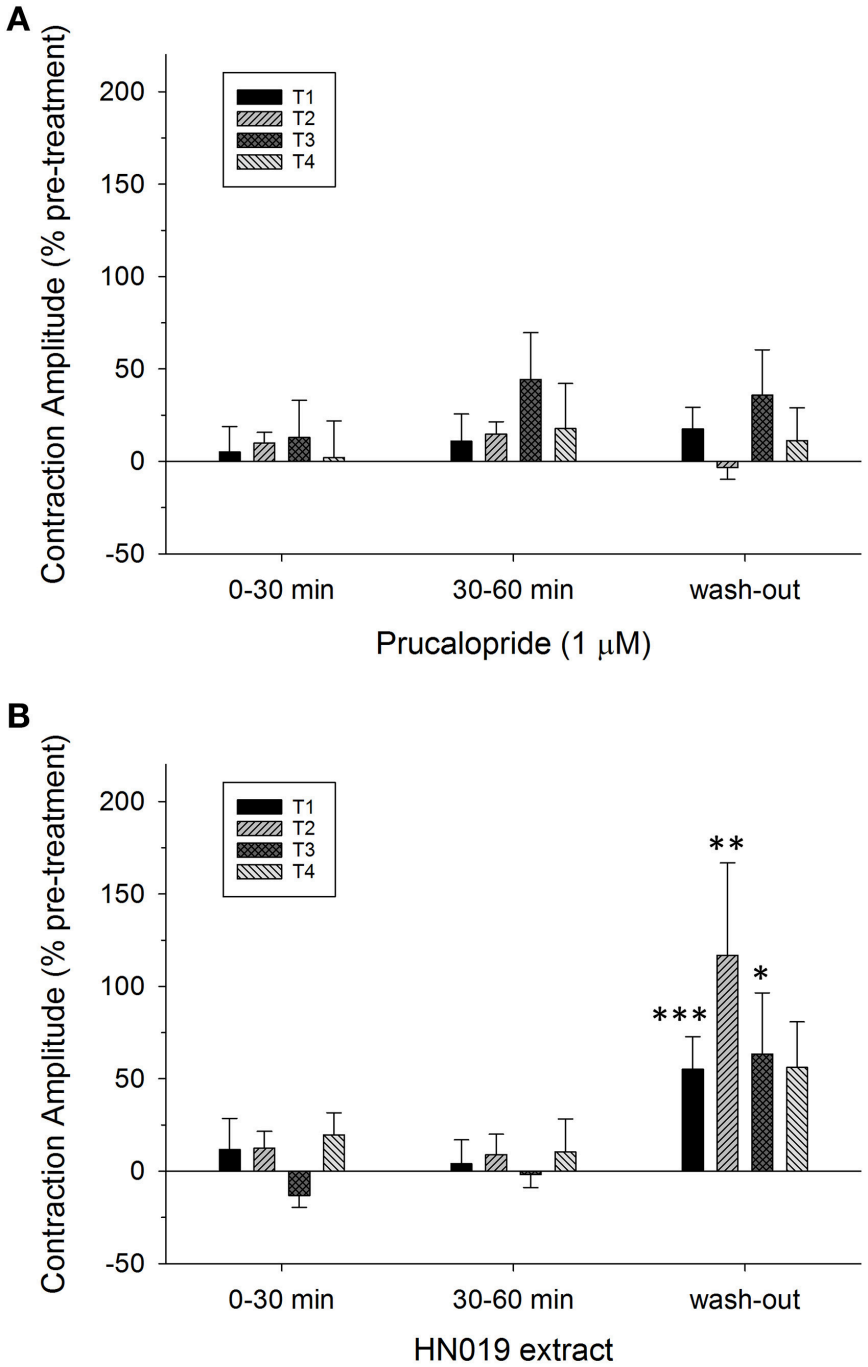
**Summary graphs of treatment effects on amplitude of synchronous contractions in the isolated large intestine**. Data are shown as percent change in contractile amplitude from the pre-treatment control at all four locations (T1, proximal; T2, mid; T3, distal; T4, rectal) for treatment conditions of: **(A)** 1 μM prucalopride (*n* = 10), and **(B)** HN019 extract (10%) (*n* = 8), and following 60 min of washout with Krebs buffer. Statistical significance was determined using repeated measures ANOVA and treatments compared with Krebs buffer treatment control (shown in Figure 2C). Data show mean ± SEM. Asterisks indicate the significance of each treatment relative to controls (^*^p < 0.05; ^**^p < 0.01; ^***^p < 0.001).

Application of 1 μM prucalopride to the large intestine tissue increased motility (Figure 1B) by increasing the frequency of propagating synchronous contractions compared with the control; by 58 ± 19% (*n* = 10) over the initial 30 min of exposure and by 75 ± 26% over 30–60 min exposure. This occurred without any change in amplitude (Figures 2A,B, 4A). The increased effects of prucalopride on contractility persisted for extended periods of time (at least 30 min following washout).

Concomitantly, prucalopride decreased the frequency of non-synchronous contractions that occurred in the proximal colon (by 50 ± 11% after 60 min, Figure 3A). Contractile amplitude was 62% less than the Krebs treatment control at 60 min (Figure 3B). These effects also persisted following wash out of prucalopride. In 2/12 preparations prucalopride inhibited synchronous contractions by 50% (these preparations only returned to 30% of pre-treatment frequency after wash out) yet tension was increased by 20–36% across the four recording sites from the mid-colon to the rectum.

### HN019 extract increased contractile amplitude after washout

Application of HN019 extract (10%) to the large intestine resulted in no detectable change in contractile activity over 60 min, yet it was found that within the first 30 min following washout of HN019, the contractile amplitude increased by 55 ± 18% in the proximal colon (Figures 1C, 2A,B). Post-exposure, HN019 extract increased the amplitude of synchronous contractions along the length of the large intestine, particularly in the distal colon (Figure 4B). In 2/10 experiments HN019 extract completely inhibited synchronous contractions during 60 min of exposure and these returned to pre-treatment levels following wash out.

HN019 extract decreased the amplitude of non-synchronous contractions in the proximal colon to 30% lower than that for the corresponding Krebs treatment control at 60 min (Figure 3B). The inhibitory effect of HN019 extract on non-synchronous contractility persisted following wash out of the treatment, with a 33% decrease in frequency and a 38% decrease in contractile amplitude compared with the Krebs treatment control.

### Ratio of non-synchronous to synchronous contractions

**Figure 5:**
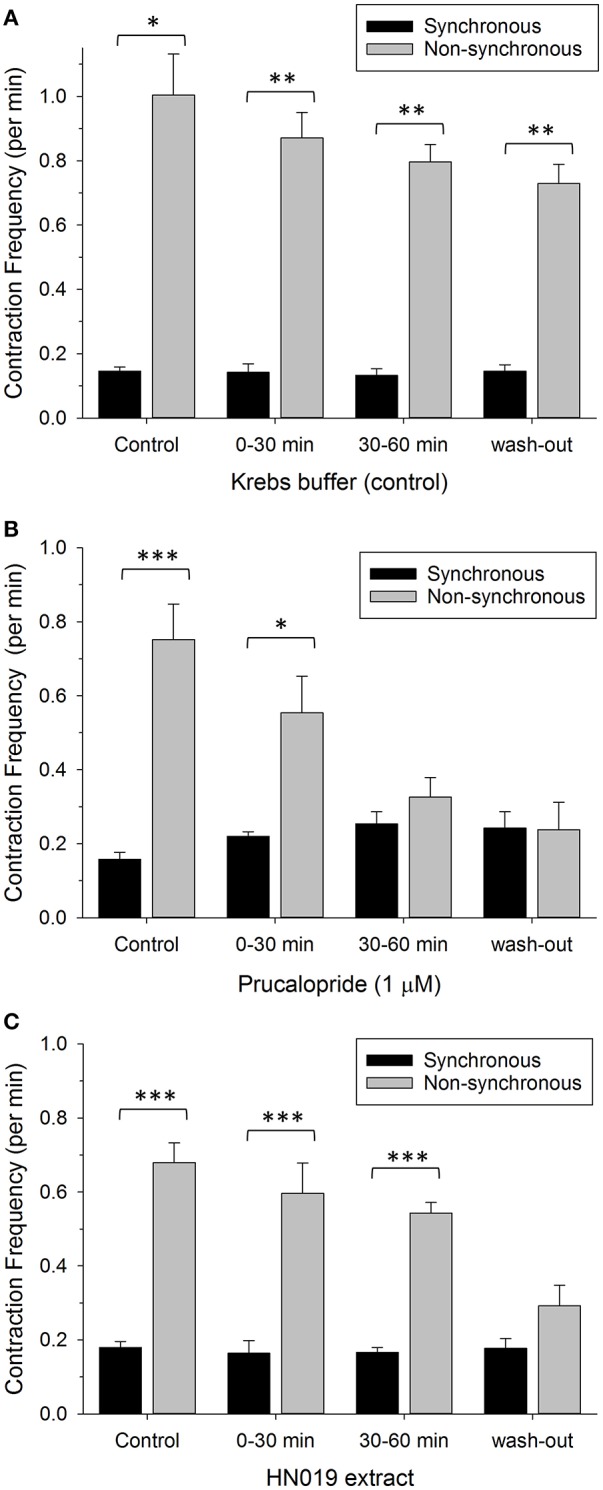
**Comparison of contractile frequency for synchronous (black) and non-synchronous (gray) contractions measured in the proximal colon**. Data are shown as number of contractions per minute for pre-treatment, treatment condition of: **(A)** Krebs buffer treatment control (*n* = 8), **(B)** 1 μM prucalopride (*n* = 10), or **(C)** HN019 extract (10%) (*n* = 8), and following 60 min of washout with Krebs buffer. Statistical significance was determined using repeated measures ANOVA and treatments compared with the Krebs buffer treatment control. Data show mean ± SEM. Asterisks indicate the significance of each treatment relative to controls (^*^p < 0.05; ^**^p < 0.01; ^***^p < 0.001).

The ratio of non-synchronous to synchronous contractions averaged 5.2:1 across the pre-treatment controls (for Krebs, prucalopride, and HN019 extract) and was reduced to 1.0:1 after 60 min of prucalopride, and 1.6:1 for HN019 extract post-treatment, yet remained high at 5.0:1 for the Krebs control (Figure 5).

## Discussion

The main findings of this study are that the predominant effect of HN019 extract was to markedly increase contractile amplitude of synchronous contractions spanning the proximal colon to the rectum, and that this effect occurred post-treatment. This supports our hypothesis that the reduced constipation effect of HN019 can be attributed to enhanced synchronous contractions in the colon. In contrast, the known prokinetic, prucalopride altered colonic motility patterns of spontaneous phasic contractions by increasing the frequency of synchronous contractions in the rat isolated large intestine both during and after treatment. The delayed effect of HN019 was unexpected and differed from prucalopride in that contractile amplitude was affected rather than frequency suggesting a different mechanism of action. Both prucalopride and HN019 extract demonstrated a secondary action in decreasing the proportion of non-synchronized to synchronized contractions. These findings may provide important insights for human patients that report altered large intestine motility habits upon consumption of these agents.

The synchronous contractions recorded were similar to those reported previously using this method (Costa et al., 2013; Dalziel et al., 2014, 2015). The continuity of motility parameters over the duration of the Krebs treatment control experiment provided a stable comparison with treatment experiments. Although a time-dependent decrease in the frequency of non-synchronous contractions was detected with Krebs treatment, this was markedly less than the main treatment effects for prucalopride and HN019 extract.

### Prucalopride

In the presence of prucalopride there was an increase in the proportion of contractions in the proximal colon that became synchronous along the length of the large intestine. Concurrently, there was a decrease in the frequency of isolated contractions in the proximal colon. This finding is consistent with its action as a promotility modulator in the colon and supports its anti-constipation effects *in vivo* (Bouras et al., 1999).

Our finding that prucalopride increased the frequency of synchronous contractions would be consistent with a coordinated firing of enteric neurons in the enteric nervous system (Yu et al., 2015). The concurrent decrease in the frequency of non-propagating contractions was surprising and the mechanisms responsible are unclear. Altered motility patterns in which their generation is dependent upon enteric ganglia have been reported for prucalopride using spatiotemporal mapping in which mid- and distal colon contractions are increased yet full length synchronous contractions are decreased in both length and frequency (Yu et al., 2015). While our method does not have the resolution to directly compare frequency with this previous study, we note a common effect of prucalopride increasing coordinated distal colon contractions.

5-HT4 receptors are expressed throughout the GI tract on numerous cell types, such as enterochromaffin cells and intrinsic primary afferent neurons, including different functional types of myenteric neurons and smooth muscle cells (De Maeyer et al., 2008). Prucalopride could therefore act at multiple sites to modulate regional and/or time-coordinated relaxation-contraction mechanisms of GI motility patterns (De Maeyer et al., 2008). Although 5-HT4 receptors are predominately expressed in the rat myenteric ganglia neurons, they are also expressed at low levels in colonic circular muscle and very low levels in longitudinal muscle and in the interstitial cells of Cajal (Poole et al., 2006). Therefore, a direct effect of prucalopride on 5-HT4 receptors in isolated colonic preparations is clearly a possibility.

Because the 5-HT4 receptor is a G-protein coupled receptor, agonist binding leads to protein kinase A activation resulting in a prolonged excitatory response (Costedio et al., 2007). In the case of myenteric cholinergic excitatory neurons, this leads to increased acetylcholine release and increased contraction (De Maeyer et al., 2008). However, the inhibitory effect of prucalopride on non-synchronous contractions in the proximal colon was slow in onset and long-lasting, suggesting a secondary effect on cellular signaling. The decrease in frequency in the Krebs treatment control suggests that prucalopride may have accelerated a time-dependent process. Another potential reason for variation in the effect of prucalopride on motility is that it is more potent on the 5-HT4b splice variant than the 5-HT4a (Pindon et al., 2002). How such an effect would alter motility patterns is unclear.

### *B. lactis* HN019

The enhanced motility effects demonstrated for HN019 extract suggest involvement by the enteric neural circuitry responsible for propulsive neurogenic colonic patterns by increasing the amplitude of propagating contractions in the colon. Further study is required to corroborate this finding and determine the underlying mechanisms involved. Thus, HN019, a probiotic known to reduce constipation increased the strength of colonic contractions in the isolated large intestine *ex vivo*. Our finding that HN019 extract also decreased non-propagating contractions suggests at least two separate effects.

The post-treatment effect of HN019 extract at increasing the strength of colonic contractions showed a considerable delay. The mechanism underlying this response is not clear and may be a consequence of removal of the extract rather than a gradual excitatory effect over time. Given that HN019 extract is a mixture of components, multiple actions would be anticipated.

The inhibitory effect of HN019 extract on non-synchronized contractions in the proximal colon was similar to that for prucalopride. Synchronous contractions that propagate along the length almost certainly require enteric neural activity and, consistent with this idea, are abolished by nerve conduction blockers (Costa et al., 2013; Dalziel et al., 2014), while those that do not propagate large distances are often of non-neural origin (Dalziel et al., 2014) in this preparation. These non-propagating contractions are generated by the interstitial cells of Cajal (ICC) (Huizinga et al., 2014). Thus, it is tempting to speculate that HN019 extract and prucalopride have a similar mechanism of action to decrease the frequency of non-propagating contractions. Experiments using tetrodotoxon to silence enteric neurons would demonstrate whether HN019 extract affects the musculature or the ICC directly.

Given that our study was *ex vivo* using serosal application of bacterial extract, the results assume that the bacterial secreted products would be absorbed and reach the peripheral circulation to be active at modulating colonic motility.

A previous study showed that HN019 given as live bacteria is effective at reducing total transit time in slightly constipated subjects (stool type of 2–4 on the Bristol Stool Chart and 1–3 bowel movements per week) (Waller et al., 2011). Our study reveals that lysed HN019 contents are sufficient to modify contractile patterns in the colon that fit a pro-motility profile of increased contractile amplitude. The mechanism by which this occurs is unclear. The ability to improve the synchrony of contractions by decreasing dysmotility might explain why HN019 is also able to reduce the severity of weanling diarrhea associated with rotavirus and *E. coli* in a piglet model over the first 3 days post-weaning (Shu et al., 2001). A related strain (GCL2505) of the *Bifidobacterium lactis* species has recently been reported to slightly increase fecal output in a rat model of constipation induced by loperamide (Aoki et al., 2016).

## Conclusion

This study supports a known prokinetic action for prucalopride that was demonstrated by an increased frequency of synchronized contractions in the proximal colon. This result could explain the known increase in colonic transit induced by prucalopride, often reported as increased emptying in the proximal colon. The change in the contractile patterns we report for prucalopride provides a benchmark for improved colonic transit against which the probiotic HN019 extract was compared.

A major finding of this study was that HN019 bacterial extract produced a delayed increase in the tension of synchronized contractions with concomitant inhibition of non-synchronized proximal colon contractions. Thus, contents from lysed *B. lactis* HN019 bacteria demonstrated prokinetic activity by increasing contractile tension. These findings provide important insights into the potential colonic actions of *B. lactis* HN019 relevant to the treatment of constipation.

## Author contributions

JED designed the study, analyzed the data, interpreted the results and wrote the paper; NS contributed pharmacological advice; JP performed the research. AL undertook the prucalopride research as a Palmerston North Medical Research Foundation Summer Scholar. RA and JD contributed microbial advice; NS, RA, JD, and NR contributed to experimental design and critically revised the manuscript.

## Funding

This work was supported by AgResearch Core funding (A18043). AL was supported by a Palmerston North Medical Research Foundation Summer Scholarship. Experiments in this project were in part conducted in the laboratory of NS and funded by the NH&MRC of Australia (#1067335).

### Conflict of interest statement

The authors declare that the research was conducted in the absence of any commercial or financial relationships that could be construed as a potential conflict of interest.
